# Supplementary material for: Monoclonal antibody levels and protection from COVID-19
Source: Nat Commun. 2023 Jul 28;14:4545. doi: 10.1038/s41467-023-40204-1 (PMC10382502; doi:10.1038/s41467-023-40204-1)
Supplement: Supplementary file 1 — Supplementary Information [file 41467_2023_40204_MOESM1_ESM.pdf]

Supplementary Information

Supplementary Methods

Meta-analysis of monoclonal antibody IC50 and neutralization titer for convalescent sera

Here we performed a meta-analysis of IC50 estimates for a range of monoclonal antibodies using data compiled in the Stanford University Coronavirus Antiviral & Resistance Database (https://covdb.stanford.edu/)[1]. In particular, all available in vitro IC50 data for approved monoclonal antibodies (and adintrevimab) was extracted from the Stanford database (on 17 January 2023). In addition, in all instances where the geometric mean neutralizing titer (GMT) of a panel of convalescent sera samples was included in one of these studies and available in the database, this data was also extracted. A number of errors were observed in the extracted limits of detection for some of these studies, and thus these were amended by re-extracting the limit of detection data from the original papers. Other minor amendments were made to the extracted data and these amendments are summarized in a supplementary file (Supplementary file, "Stanford-approved mAbs and adintrevimab -2023-01-17_Updated.csv"). For convalescent sera, only data for individuals infected with "Wild Type" SARS-CoV-2 and with sera assessed 1 month (i.e. indicated "1m" in database) after infection were included in the meta-regression. The overall IC50 for each combination of monoclonal antibody and variant was estimated by fitting a linear mixed effects model with censoring of IC50 values above 10,000 ng/ml (using the lmec package in the statistical software, R[2, 3]). Only the following mAb and combinations were considered:

> Bamlanivimab + Etesevimab, Bamlanivimab, Etesevimab, Casirivimab + Imdevimab, Casirivimab, Imdevimab, Cilgavimab + Tixagevimab, Cilgavimab, Tixagevimab, Amubarvimab + Romlusevimab, Amubarvimab, Romlusevimab, Adintrevimab, Regdanvimab, Sotrovimab, Bebtelovimab, Bamlanivimab + Bebtelovimab + Etesevimab.

An estimate was only calculated for mAb against a variant if at least three studies were available that reported an IC50 against that variant. For mAbs, the negative log of the IC50 value was used in the regression, and for convalescent sera the log of the GMT were used in the regression. Values below -log(10,000) were censored (i.e. negative log of the upper IC50 limit of detection (LOD) of most studies which was 10,000 ng/ml). In the instances where a study reported a higher LOD than the 10,000 ng/ml usually used, censoring of these studies was still performed at 10,000 ng/ml, and in the

studies where the LOD tested was below 10,000 ng/ml, and when a study also found an IC50 for a mAb was above the LOD, these values were excluded from the analysis. The regression model used is given by:

$$Y_{50} \sim \text{Variant} * \text{mAb} + (1 \mid \text{StudyAssay}), \qquad \text{(Eq. S1)}$$

where Y_{50} is the negative log transformation of the IC50, "Variant" is a covariate for the variant against which IC50 was assessed in the study, and mAb is a covariate describing which monoclonal antibody, antibody combination or convalescent plasma was being assessed. Inter-study variability was accounted for using a random effect for each individual assay reported in each study (StudyAssay) applied to the intercept of the regression. The random effect variant was 0.86.

Extraction of case data and efficacy with 95% confidence bounds

We extracted data of the number of cases in treatment and control groups at different time intervals after administration of treatment from five studies. The source for the case data and the frequency and duration of time intervals for which cases could be extracted are included in the overview of studies (**Table S1**). Data was extracted independently by two authors (ES and SRK), and discrepancies were resolved through discussion – all discrepancies were minor and were resolved through mutual agreement by the extractors that one of the extraction attempts contained an error. Data was extracted from figures using WebPlotDigitizer[4] or Adobe Illustrator[5].

For the studies by Isa et al.[6] and Levin et al.[7], the number of subjects at risk in the different reported time intervals in the placebo and the treatment group could be extracted along with the number of cases (symptomatic infections). In the studies by O'Brien et al.[8] and Herman et al.[9], the number of subjects at risk in the different time intervals could not be extracted. For these studies, we assumed that the number of subjects at risk reduced by the number of events in previous time intervals, i.e. we assumed the effect of subjects being lost to follow-up is negligible compared to case numbers.

For the study by Schmidt et al.[10], the number of subjects at risk was not provided. We used the number of symptomatic infections and percentage of the subjects at risk to estimate the number of subjects at risk (rounded to the nearest integer).

We computed the efficacy and confidence intervals at each time interval (**Table S1**) from the number of events and subjects at risk for the treatment and control group in that interval. Efficacy was estimated as 1 – relative risk (as reported previously[11]), i.e.

$$1-\frac{\text{number events in treatment group/number of subjects in treatment group}}{\text{number events in control group/number of subjects in control group}}. \qquad \text{(Eq. S2)}$$

To visualize the uncertainty in efficacy estimates, we computed the 95% confidence intervals for the efficacy estimates from the extracted data using the Katz-log method specified in supplementary table 1 of Aho & Bowyer[12] (as previously[13]). Thus, the 95% confidence intervals for the efficacy are

$$\left[1-\frac{\frac{e_t}{n_t}}{\frac{e_c}{n_c}}\times\exp\left(\pm 1.96\times\sqrt{\frac{1}{e_t}+\frac{1}{e_c}-\frac{1}{n_t}-\frac{1}{n_c}}\right)\right], \qquad \text{if } e_t>0 \text{ and } e_c>0, \qquad \text{(Eq. S3)}$$

$$\left[1-\frac{(e_t+0.5)/n_t}{e_c/n_c}\times\exp\left(1.96\times\sqrt{\frac{1}{e_t+0.5}+\frac{1}{e_c}-\frac{1}{n_t}-\frac{1}{n_c}}\right),1\right], \qquad \text{if } e_t=0, \qquad \text{(Eq. S4)}$$

$$\left[1-\frac{e_t/n_t}{(e_c+0.5)/n_c}\times\exp\left(\pm 1.96\times\sqrt{\frac{1}{e_t}+\frac{1}{e_c+0.5}-\frac{1}{n_t}-\frac{1}{n_c}}\right)\right], \qquad \text{if } e_c=0, \qquad \text{(Eq. S5)}$$

where e_t and e_c are the numbers of events (symptomatic infections) in the treatment and control groups respectively and n_t and n_c are the numbers of subjects at risk in the treatment and the control group respectively. If this expression is negative, then the lower bound is set to 0. If there are 0 events in both the treatment and control group, then the efficacy and confidence intervals are not defined at that time interval. Importantly, these confidence intervals are only used for visualization of efficacy data, and when data fitting was performed the raw event data was used in the analysis.

Extraction of concentration data and estimating the geometric mean concentration for given time intervals

We also extracted mAb concentration data from the different studies (**Table S1**) using WebPlotDigitizer[4] or Adobe Illustrator[5]. For the studies by Isa et al.[6], Herman et al.[9] and Levin et al.[7], we extracted an estimate of the total concentration of both antibodies used in combination (i.e. casirivimab + imdevimab or tixagevimab + cilgavimab, respectively). For the study by O'Brien et al.[8]

we extracted the concentration of casirivimab and imdevimab separately and added these concentrations together to estimate the total concentration of both casirivimab and imdevimab. For the study by Schmidt et al.[10], we extracted the modelled concentration data, since the raw concentration data were visually obscured due to the high number of overlapping concentration data points. As with the clinical data, all antibody concentration data was extracted independently by two separate authors (ES and SRK) and all extracted values very similar between the two extractors (no difference greater than 0.11 of a $\log_{10}$). The geometric mean of the two extractions was used in the final analysis.

We estimated the geometric mean concentration for each time interval over which efficacy data was available by first linearly interpolating between the (log) concentration reported at each time point in the extracted concentration data (using the function *approx* in R[14]). The geometric mean concentration for each time interval was computed as,

$$\exp\left(\frac{\int_{t_{min}}^{t_{max}} \log(c(t))\, dt}{t_{max} - t_{min}}\right), \qquad \text{(Eq. S6)}$$

where $c(t)$ is the linearly interpolated (log) concentration by time and t_{min} and t_{max} are the lower and upper end of the time interval. The geometric mean concentration was computed for each study and each time interval of case data (see horizontal error bars in **Figure 1**).

Test of concentration effect on the efficacy of prophylactic mAb treatment

We tested if there is a significant association between antibody concentration and efficacy in these data. For this analysis, we used a log-binomial regression model, a generalized linear mixed model (GLMM) with a binomial error family and logarithmic link function (using the *glmer* function of the *lme4* package[15] in R version 4.2.1[14]). The model includes random intercepts for different trials, the covariate "treatment" and the interaction of treatment with concentration. The model is described by

$$(E, NE) \sim 1 + \text{Treatment} + \text{treatment} : \log_{10}(\text{concentration}) + (1 \mid \text{Trial}), \qquad \text{(Eq. S7)}$$

where *E* is the number of events (symptomatic infections) and *NE* is the number of subjects without an event (i.e., no symptomatic infection), the *Treatment* variable indicates the treatment group (1) or control subgroup (0), ":" indicates an interaction term, the *concentration* is the geometric mean concentration, and *Study* indicates the study. The significance of the interaction of treatment and concentration was tested with a chi-squared test (with the function *drop1*).

Multiple imputation of mAb concentration and in vitro IC50 data

To take into consideration variability in the mAb concentration and the in vitro IC50 (i.e. x-axis uncertainty) in fitting the dose-response relationship, we used multiple imputation of the x-position of each data point in Figure 2, i.e. the concentration data and in vitro IC50s. In particular, a single imputation for a given data point (i.e. efficacy estimate associated with a particular time interval, Table S1) was conducted by randomly sampling the day from the data points time interval (assuming a uniform distibrution). We then estimated the geometric mean mAb concentration observed on this randomly sampled day (the mAb concentration was linearly interpolated between the available antibody concentration time points reported in each study). Since, the antibody concentrations at each time interval, were also normalized by the in vitro IC50 for each antibody from our meta-regression, and since these IC50 estimates contain uncertainty, we also randomly sampled the estimated in vitro IC50 from our meta-analysis, by sampling from a normal distribution where the mean is the (log) of the estimated IC50 and the standard deviation is the standard error of the (log) of the IC50 estimate from the meta-regression. The sampled log-IC50 was then exponentiated. The fold-IC50 concentration in each imputation was calculated as

$$\text{concentration [fold-IC50]}=\frac{\text{concentration [mg/L]}}{\text{IC50 [ng/mL]} \times 10^{-3}}. \quad \text{(Eq. S8)}$$

At each imputation, we then fitted the dose-response curve using the same procedure outlined in the primary analysis (see Methods). Overall, we imputed 100 data sets and combined the estimates for the parameters of the dose-response curve using Rubin's rules[16].

Thus, the estimate for the parameter vector $\overline{\mathbf{p}}$ is

$$\bar{\mathbf{p}} = \frac{1}{n}\sum_{j=1}^{n} \hat{p}_j, \qquad \text{(Eq. S9)}$$

where n is the number of imputed data sets and $\hat{p}_j$ is the vector of parameter values estimated from the *j*th imputed data set. The estimated within imputation variance is given by

$$W = \frac{1}{n}\sum_{j=1}^{n} W_j, \qquad \text{(Eq. S10)}$$

where W_j is the variance-covariance matrix of the parameter estimate $\hat{p}_j$ for the *j*th imputed data set (which was as an output of the model fitting using nlm in R). The estimate for the variance between the $n = 100$ parameter estimates is

$$B = \frac{1}{n-1}\sum_{j=1}^{n} (\hat{p}_j - \bar{p})(\hat{p}_j - \bar{p})'. \qquad \text{(Eq. S11)}$$

Finally, the estimate for the total variance of the parameters is

$$T = W + \left(1 + \frac{1}{n}\right) B. \qquad \text{(Eq. S12)}$$

We found that the variance within imputations ($W = 10^{-2} \times \begin{pmatrix} 3.2 & 6.4 \\ 6.4 & 30.7 \end{pmatrix}$) is much larger than the variance between imputations ($B = 10^{-11} \times \begin{pmatrix} 8.1 & 19.2 \\ 19.2 & 66.8 \end{pmatrix}$). This means that the variation between bootstraps is very small compared to the uncertainty of the dose-response curve parameters for each bootstrapped data set, i.e. the uncertainty in the x-position (the dose) appears to be small compared to the error in the y-position (the efficacy estimate). Due to low numbers of events, the uncertainty in the efficacy estimate is large in some cases (see the confidence intervals for the efficacy in Figure 1 and Figure 2). Thus, the total variance is very similar to the within imputation variance and the estimated parameter values and their 95% confidence intervals are almost exactly the same as for the parameter estimate to the original data (Table S6 and Table S7).

Estimating antibody half-life

In order to predict the duration of protection of each monoclonal antibody combination, we first estimated the *in vivo* half-life for casirivimab + imdevimab, cilgavimab + tixagevimab, and adintrevimab. The half-lives were estimated by fitting a linear regression model to the log-transformed concentration data from the time of the peak antibody concentration onwards (Figure S3). This fitting was performed using the *lm* function in R and the function *confint* to determine 95% confidence intervals for the estimated half-lives. We found that the estimated half-lives agree well with the mean of the half-lives of casirivimab and imdevimab or tixagevimab and cilgavimab that were reported in the literature (Table S8).

Duration of protection

Using the dose-response curve, we can predict the protection over time and how long the protection remains above 50% protection (Figure 3). With the meta-analysis of IC50's against different variants, the duration of protection can be predicted not only for the ancestral strain but also for variants (Figure 3). We assumed that the concentration of mAbs declines exponentially from the time of the peak concentration for each antibody (extracted from the data) with a half-life fitted to the data (linear fit to log-transformed concentration, see Table S8). We first normalized the concentration over time data and dividing this by the IC50s of each antibody against each variant. We then used these normalized antibody concentrations and the relationship between efficacy and normalized concentration (in Figure 2) to compute the protection over time. We used this procedure to compute the time until efficacy dropped below 50%. The uncertainty in the time to 50% protection is due to the uncertainty in the concentration that gives 50% protection (Table S6). The upper and lower bounds for the time to 50% protection are the time to reach the upper or lower bound of the 95% CI of the concentration that gives 50% protection, respectively.

Comparison of the relationship between neutralizing antibodies and protection for vaccination and monoclonal antibody prophylaxis

To compare the mAb data with the data from vaccine studies (Figure 4a), we aimed to match the mAb data as closely as possible with the vaccine studies. Thus, we restricted the mAb data to 2-3 months after treatment, subjects who are PCR-negative at baseline, and cases later than 1-2 weeks after treatment (the start of follow-up in vaccine studies) (Figure 4a). We then used a generalized linear mixed model (GLMM) with a binomial error family and logarithmic link function (see above).

The model included random intercepts for different trials and a treatment variable with the factors “control”, “mAb” and “vaccine”. The treatment effect of mAbs and vaccination was compared by testing if there is a significant difference between the coefficients for mAb treatment and vaccination (using the *glht* function from the *multcomp* package[17]).

To further compare the efficacy of vaccination and prophylactic mAb treatment, we normalized the concentration to a common ‘fold-convalescent’-scale (as previously described[11, 13]). Using a maximum likelihood approach (see above), we fitted logistic dose-response curves to the monoclonal antibody and vaccine data (Figure 4 and Figure S5). We tested whether there is a significant difference between the monoclonal antibody prophylaxis and vaccination by fitting all data with the same parameters for the two types of treatment (Figure S5a) and compared this fit to fits which have different parameters for antibody treatment and vaccination (e.g., Figure 4b and Figure S5b-d red and blue curves). Different models were compared using AICs and with a likelihood ratio test. The p-values for different model comparisons are reported in Figure S4 and parameter values for different fits in Table S9. Confidence intervals were calculated using the Hessian of the parameter fit, as described in the Methods.

Supplementary Tables

Overview of prophylactic mAb studies

Trial	Ref.	mAb	Dose [mg]	Admin.	Notes/Study description	Case data source	Duration of follow-up used in analysis (days post administration)	Time intervals for which cases could be extracted (days post administration)	Antibody concentration data source
**Isa**	6	casirivimab + imdevimab	1,200	SC	Administered every 4 weeks for up to 6 doses	Figure 1	28 – 167	1-27*, 28-55, 56-83, 84-111, 112-139*, 140-167, 168-196*	Figure 2
**O'Brien**	8	casirivimab + imdevimab	1,200	SC	Treatment of household contacts of confirmed cases<br>Subgroup of seronegative at baseline used here	Table S4	8 – 28	1-7◊, 8-14, 15-21, 22-28	Figure S4
**Herman**	9	casirivimab + imdevimab	1,200	SC	Same as O'Brien study but with extended follow-up (8 months)	Figure 2A	31 – 240	1-30◊, 31-60, 61-90, 91-120, 121-150, 151-180, 181-210, 211-240	Table S6
**Levin (PROVENT)**	7	tixagevimab + cilgavimab	300	IM	Treatment of high-risk subjects (inadequate response to vaccination and/or high exposure)	Figure 2	11 – 183	0-10#, 11-29, 30-59, 60-89, 90-119, 120-149, 150-179, 180-183	Figure S2A
**Schmidt**	10	adintrevimab	300	IM	Phase 2/3 clinical study of efficacy during the Delta and Omicron BA.1/BA.1.1 waves	Table S3	Delta: 91 – 180<br>Omicron: 29 – 90	Delta: 1-90◊, 91-180<br>Omicron: 1-28◊, 29-60, 61-90	Figure 2A (model)

Table S1 Overview of identified prophylactic mAb studies that are used in the analysis. The earliest cases were excluded for all studies (note that for the Isa study there were no cases in either the treatment or the control group in the first 28 days after treatment). Abbreviations: SC subcutaneous, IM intramuscular, IV intravenous. * For these time intervals, there were no cases in both the treatment and the control group, thus the efficacy could not be computed, and the time intervals were excluded. # For the earliest time interval in the study by Levin et al., the geometric mean concentration could not be computed as the first data point for the *in vivo* concentration is on day 8. We excluded this time interval from the analysis. ◊ The earliest time intervals in the O'Brien et al., Herman et al., and Schmidt et al. studies are shown in **Figure 2** but were excluded from the analysis (see Methods).

Additional information on prophylactic mAb studies

Trial	Ref.	Source of mAb concentration data (all/subset of participants)	Seronegative participants at baseline	Vaccination of participants during trial	Circulating variant(s) during the trial	Variant used for analysis
**Isa**	6	All	Total: 85.1%<br>Treatment group: 84.6%<br>Placebo group: 86.7%	Discontinued from study drug and entered follow-up if elected to receive COVID-19 vaccine	Not VOC*	Wild Type
**O'Brien**	8	Subset	100%	Excluded if received a SARS-CoV-2 vaccine	NR	Wild Type
**Herman**	9	Subset	100%	Vaccination prohibited before randomization, but allowed during follow-up (similar rates of vaccination in control and treatment arms (35.2% vs 34.5%, with total of 3 cases in individuals after vaccination)	Not VOC	Wild Type
**Levin (PROVENT)**	7	Subset	100%	Excluded if received a SARS-CoV-2 vaccine	Not VOC (9/20)<br>Alpha (5/20)<br>Beta (1/20)<br>Delta (5/20)	Wild Type#
**Schmidt**	10	Model of the mAb concentration measured in a subset of participants	100%	Excluded if received a SARS-CoV-2 vaccine	Alpha<br>Delta<br>Omicron (BA.1, BA1.1, BA.2)	Delta (pre-Omicron period)<br>Omicron BA.1 (post-Omicron period)+

Table S2 Additional information on the prophylactic mAb studies used in the analysis. Abbreviations: NR not reported, VOC variant of concern. # Cases were most commonly infected with a variant that is not a variant of concern (9 out of 20 sequenced variants, 45%, Table S11 of reference). Thus, we used the wild type variant for our analysis. + The majority of cases in the pre-Omicron data were due to the Delta variant (97.7%, Table S2 of reference). In the post-Omicron data, most cases were infected with the Omicron BA.1 variant (61.9%, Table S2 of reference).

Estimated in vitro IC50 for monoclonal antibodies (and geometric mean neutralization titer of convalescent plasma) against ancestral virus and Omicron subvariants from systematic review and meta-analysis

Variant	Adintrevimab	Casirivimab/ Imdevimab	Cilgavimab/ Tixagevimab	Convalescent Titer*
Wild Type	5.46 (3.72, 8.03)	4.12 (2.95, 5.75)	4.27 (3.1, 5.88)	561 (861, 366)
Alpha	5.46 (2.41, 12.3)	2.49 (1.31, 4.74)	4.3 (2.19, 8.43)	NC
Beta	10.9 (4.85, 24.7)	9.13 (5.36, 15.6)	10.9 (5.91, 20.1)	NC
Delta	8.02 (4.22, 15.3)	7.22 (4.08, 12.8)	5.87 (3.51, 9.83)	NC
Gamma	8.06 (3.57, 18.2)	6.98 (3.27, 14.9)	4.58 (2.24, 9.35)	NC
Omicron BA.1	498 (313, 791)	>10000 (8670, >10000)	270 (184, 394)	NC
Omicron BA.1.1	620 (306, 1250)	NC	524 (257, 1070)	NC
Omicron BA.2	>10000 (>10000, >10000)	1660 (1010, 2740)	38.2 (25.8, 56.4)	NC
Omicron BA.2.12.1	>10000 (1.45, >10000)	1110 (550, 2240)	31.9 (16.4, 62.1)	NC
Omicron BA.2.75	>10000 (0.043, >10000)	2200 (911, 5320)	90.6 (44.8, 184)	NC
Omicron BA.2.75.2	NC	>10000 (0.0126, >10000)	>10000 (0.0126, >10000)	NC
Omicron BA.3	>10000 (9110, >10000)	NC	108 (40.4, 287)	NC
Omicron BA.4.6	NC	4000 (1290, >10000)	>10000 (7720, >10000)	NC
Omicron BA.5	>10000 (>10000, >10000)	1640 (946, 2840)	121 (79.4, 185)	NC
Omicron BQ.1	NC	NC	>10000 (0.0003, >10000)	NC
Omicron BQ.1.1	NC	>10000 (0.0126, >10000)	>10000 (0.156, >10000)	NC
Omicron XBB	NC	NC	>10000 (0.0003, >10000)	NC

Table S3 The geometric mean IC50 in ng/mL (95% Confidence Intervals) for monoclonal antibodies estimated using a meta-analysis of data from the Stanford University Coronavirus Antiviral & Resistance Database (https://covdb.stanford.edu/)[1] . Abbreviations: NC: Not computed. The meta-regression was performed on the log-transformed IC50 values reported for each antibody variant combination using a linear mixed effects model with a random effect for study/assay and censoring of IC50's above 10,000 ng/ml. Where the estimated geometric mean IC50 was above 10,000 the value reported is ">10,000". *Where studies included a panel of convalescent serum assessed for neutralization in the same study using the same assay as that used for the IC50 (n=19 included this information), the mean titer reported by the study was extracted and the inverse of this titer was included in the meta-regression.

Relationship between mAb concentration and efficacy

Data						P-value (Wald test for slope parameter)
O'Brien	Herman	Isa	Levin	Schmidt (Delta)	Schmidt (Omicron)	
All data (6 study-variant combinations)						
1	1	1	1	1	1	<0.0001
5 study-variant combinations (leave one out)						
1	1	1	1	1	0	0.0020
1	1	1	1	0	1	<0.0001
1	1	1	0	1	1	<0.0001
1	1	0	1	1	1	<0.0001
1	0	1	1	1	1	<0.0001
0	1	1	1	1	1	<0.0001
4 study-variant combinations (leave two out)						
1	1	1	1	0	0	0.0030
1	1	1	0	1	0	0.0020
1	1	1	0	0	1	<0.0001
1	1	0	1	1	0	0.0019
1	1	0	1	0	1	<0.0001
1	1	0	0	1	1	<0.0001
1	0	1	1	1	0	0.2996
1	0	1	1	0	1	<0.0001
1	0	1	0	1	1	<0.0001
1	0	0	1	1	1	<0.0001
0	1	1	1	1	0	0.0031
0	1	1	1	0	1	<0.0001
0	1	1	0	1	1	<0.0001
0	1	0	1	1	1	<0.0001
0	0	1	1	1	1	<0.0001
3 study-variant combinations (leave three out)						
1	1	1	0	0	0	0.0071
1	1	0	1	0	0	0.0019
1	1	0	0	1	0	0.0017
1	1	0	0	0	1	<0.0001
1	0	1	1	0	0	0.5006
1	0	1	0	1	0	0.3932
1	0	1	0	0	1	<0.0001
1	0	0	1	1	0	0.3236
1	0	0	1	0	1	<0.0001
1	0	0	0	1	1	0.0001
0	1	1	1	0	0	0.0043
0	1	1	0	1	0	0.0030
0	1	1	0	0	1	<0.0001
0	1	0	1	1	0	0.0031

0	1	0	1	0	1	<0.0001
0	1	0	0	1	1	<0.0001
0	0	1	1	1	0	0.4056
0	0	1	1	0	1	<0.0001
0	0	1	0	1	1	0.0001
0	0	0	1	1	1	0.0001
2 study-variant combinations (leave four out)						
1	1	0	0	0	0	0.0033
1	0	1	0	0	0	0.8701
1	0	0	1	0	0	0.4595
1	0	0	0	1	0	0.3527
1	0	0	0	0	1	0.0002
0	1	1	0	0	0	0.0110
0	1	0	1	0	0	0.0017
0	1	0	0	1	0	0.0017
0	1	0	0	0	1	<0.0001
0	0	1	1	0	0	0.6199
0	0	1	0	1	0	0.4689
0	0	1	0	0	1	0.0002
0	0	0	1	1	0	0.6295
0	0	0	1	0	1	0.0002
0	0	0	0	1	1	0.1017

Table S4 Assessing whether there was a significant association between the efficacy and the mAb concentration (in fold-IC50) using a generalized linear mixed effects model with a binomial error family and logarithmic link function, and a chi-squared test for the significance of the mAb concentration as a covariate (two-tailed Wald test), i.e. to test for a significant slope of the efficacy with the mAb concentration (see Methods). Gray-shaded rows indicate where significance was not achieved, and only occurred when both the Herman et al., and Schmidt et al., studies were excluded.

Efficacy model comparison

Model	Model equation	Parameters	Parameter estimates	AIC	ΔAIC
**Logistic model with maximal efficacy 1**	$\frac{1}{1+\exp\left(-k\times\left(\log_{10}(c)-\log_{10}(c_{50})\right)\right)}$	k: slope parameter<br>c_{50}: concentration at which the efficacy is 50%	k: 1.3<br>c_{50}: 96.2	124.6	0
**Logistic model**	$\frac{m}{1+(2m-1)\times\exp\left(-k\times\left(\log_{10}(c)-\log_{10}(c_{50})\right)\right)}$	m: maximal efficacy<br>k: slope parameter<br>c_{50}: concentration at which the efficacy is 50%	m: 0.961<br>k: 1.6<br>C_{50}: 96.1	126.4	1.8
**Logistic model with slope 1**	$\frac{m}{1+(2m-1)\times\exp\left(-\left(\log_{10}(c)-\log_{10}(c_{50})\right)\right)}$	m: maximal efficacy<br>c_{50}: concentration at which the efficacy is 50%	m: 1.00<br>c_{50}: 58.4	125.4	0.8
**Threshold model**<br>constant efficacy below and above the threshold concentration	$\begin{cases} e_{\text{below}}, & \text{if } c < c_{\text{thr}} \\ e_{\text{above}}, & \text{else} \end{cases}$	c_{thr}: threshold concentration<br>e_{below}: efficacy below the threshold<br>e_{above}: efficacy above the threshold	c_{thr}: 1,116.3<br>e_{below}: 0.416<br>e_{above}: 0.922	125.2	0.6

**Double logistic model**<br>Logistic model with a change of the slope at the threshold concentration	$\begin{cases} \dfrac{m}{m + \exp(-k_1 \times \log_{10}(\mathrm{c}))}, & \text{if } \mathrm{c} < \mathrm{c_{thr}} \\ \dfrac{m}{m + \exp\left(-k_1 \times \log_{10}(\mathrm{c_{thr}}) - k_2 \times \left(\log_{10}(\mathrm{c}) - \log_{10}(\mathrm{c_{thr}})\right)\right)}, & \text{else} \end{cases}$	m: maximal efficacy<br>k_1, k_2: slope parameters<br>$\mathrm{c_{thr}}$: threshold concentration	m: 0.941<br>k_1: 1.4<br>k_2: 0<br>$\mathrm{c_{thr}}$: 8,363.5	128.1	3.5
**Exponential model**<br>Exponential decay of the relative risk of symptomatic infection with the $\log_{10}$-concentration of the monoclonal antibody	$1 - \exp(-\lambda \times \log_{10}(\mathrm{c}))$	λ: slope parameter	λ: 0.5	126.8	2.2

Table S5 Comparison of the fit of different efficacy models to the data. We considered six different models for the protective efficacy of prophylactic mAb treatment depending on the mAb concentration (c) and compare them using the AIC. To fit the models, 100 random initial parameters were sampled uniformly from the following parameter ranges: $k, k_1, k_2 \in [0.1, 100]$, $c_{50} \in [10, 5000]$, $m \in [0.6, 0.99]$, $c_{thr} \in [50, 35000]$, $e_{below}, e_{above} \in [0,1]$, and $\lambda \in [0, 1000]$. The best fit for each of these models is shown in **Figure S2**.

Parameter estimates for the dose-response curve

Parameter	Estimate	95% confidence interval
Slope parameter	1.3	0.9 – 1.8
Concentration that gives 50% protection [fold in vitro IC50]	96.2	32.4 – 285.2

Table S6 Parameter estimates and 95% confidence intervals for the dose-response curve of efficacy by concentration [fold in vitro IC50]. The efficacy function is a logistic function with maximal efficacy 1 (i.e. maximum efficacy = 100%). These parameters were estimated by model fitting (**Figure 2**).

Parameter estimates for the dose-response curve with imputed concentration and in vitro IC50 (accounting for x-axis uncertainty)

Parameter	Estimate	95% confidence interval
Slope parameter	1.3	0.9 – 1.8
Concentration that gives 50% protection [fold in vitro IC50]	96.2	32.5 – 285.2

Table S7 Parameter estimates and 95% confidence intervals for the dose-response curve of efficacy by concentration [fold in vitro IC50]. The efficacy function is a logistic function with maximal efficacy 1 (100%). These parameters were estimated by model fitting taking into account variability in the mAb concentration and the in vitro IC50 by imputation. For each imputation (overall, n=100 imputations), we uniformly sampled a day from the time interval for the efficacy estimate and used the mAb concentration of this day (linearly interpolated on a log-scale, see **Figure 1**). The mAb concentration was then converted to fold in vitro IC50 using a sampled IC50 (normally distributed on a log-scale, mean and standard deviation were estimated in the meta-analysis). Best fit parameters from different imputations were combined using Rubin's rules[16].

Temporal kinetics of monoclonal antibody concentrations

	Adintrevimab	Casirivimab + imdevimab	Cilgavimab + tixagevimab
Source of concentration data	Schmidt	O'Brien & Herman	Levin
Peak concentration [mg/L]	36.6	107.9	24.0
Day of peak concentration	9	3	29
Half-life estimated from the data [days]	134.4	28.9	94.7
95% CI of the estimated half-life	132.6 – 136.3	26.6 – 31.6	83.5 – 109.4
Half-life from the literature [days]	141	29.4	86.5
Reference for half-life	10	18	19

Table S8 Summary of the kinetics of the antibody concentration over time for adintrevimab, casirivimab/imdevimab and cilgavimab/tixagevimab. For casirivimab/imdevimab, only the data from the O'Brien and Herman studies was used, since individuals were re-treated every 4 weeks in the Isa study. The half-life from the data was estimated by fitting a linear model to the log-transformed concentration data from the peak (Figure S3). The half-life from the literature is the reported half-life of adintrevimab and the mean of the reported half-lives of casirivimab and imdevimab or tixagevimab and cilgavimab, respectively. Abbreviation: CI confidence interval.

Parameter values for different model fits comparing the efficacy of vaccines and prophylactic mAbs

<table>
<tr><th colspan="2">Model</th><th>k</th><th>c_{50}</th><th>Fold-difference of parameter between mAb and vaccination (95% CI)</th><th>Negative log-likelihood</th><th>AIC</th><th>ΔAIC</th></tr>
<tr><td colspan="2">same k, c_{50}</td><td>1.82</td><td>0.16</td><td>-</td><td>128.6</td><td>196.6</td><td>5.9</td></tr>
<tr><td rowspan="2">different k same c_{50}</td><td>vaccine data</td><td>2.41</td><td rowspan="2">0.21</td><td rowspan="2">k: 0.54 (0.40 – 0.75)</td><td rowspan="2">120.7</td><td rowspan="2">190.7</td><td rowspan="2">0</td></tr>
<tr><td>mAb data</td><td>1.32</td></tr>
<tr><td rowspan="2">different c_{50} same k</td><td>vaccine data</td><td rowspan="2">1.95</td><td>0.16</td><td rowspan="2">c_{50}: 2.83 (1.17 – 6.86)</td><td rowspan="2">125.6</td><td rowspan="2">195.6</td><td rowspan="2">4.9</td></tr>
<tr><td>mAb data</td><td>0.44</td></tr>
<tr><td rowspan="2">different k, c_{50}</td><td>vaccine data</td><td>2.44</td><td>0.21</td><td>k: 0.52 (0.34 – 0.78)</td><td rowspan="2">120.6</td><td rowspan="2">192.6</td><td rowspan="2">1.9</td></tr>
<tr><td>mAb data</td><td>1.27</td><td>0.17</td><td>c_{50}: 0.81 (0.26 – 2.51)</td></tr>
</table>

Table S9 Parameter values for the different model fits to compare vaccine and mAb data. The models were fit using a maximum likelihood approach and a likelihood ratio test was used to compare models (see Supplementary Methods). Fitted models and comparisons are shown in Figure 4b, Figure S4 and Figure S5. The best fitting model based on AICs and likelihood-ratio test comparisons is the model with the same concentration that gives 50% protection (c_{50}) but different slope (k) (bold line above). If there are two values in a cell for a model, the upper one is the estimate for the vaccine data and the lower is the estimate for the mAb data. If there is only one value, then the vaccine and mAb data estimates are the same. The concentration that gives 50% protection (c_{50}) is given in fold-convalescent scale here (to compare the vaccine and the mAb data the concentrations were transformed to fold-convalescent by dividing by the mean convalescent neutralization titer). The fold-difference of the parameters is the ratio of the parameter for mAbs and vaccines. Abbreviations: m maximal efficacy, k slope parameter, c_{50} concentration that gives 50% protection.

Supplementary Figures

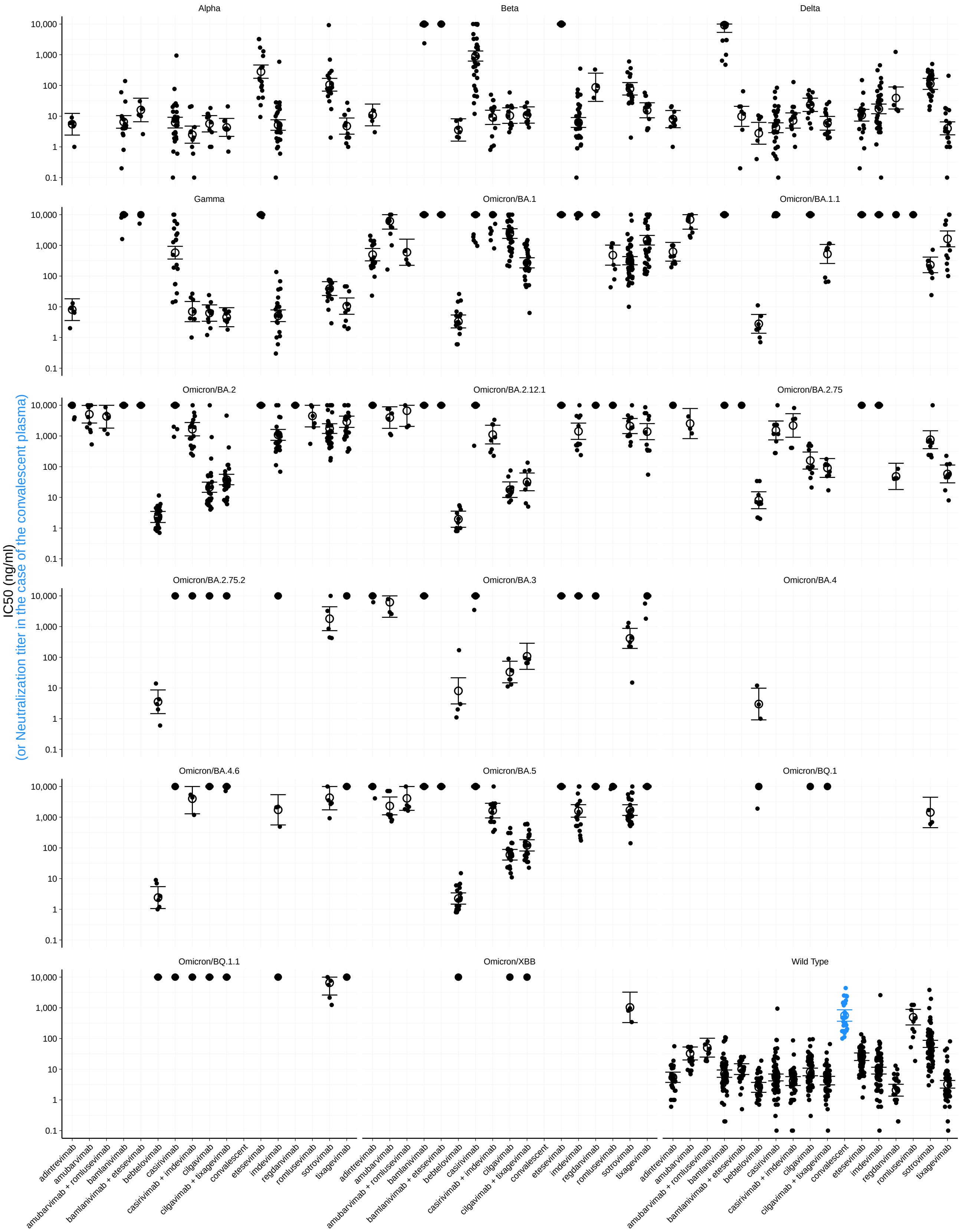

Figure S1 A boxplot of the IC50 values reported for each antibody against each variant in each study of used in the meta-regression of data from the Stanford University Coronavirus and Resistance database (https://covdb.stanford.edu/)[1]. Each small dot represents the reported IC50 from an individual study and the large open circles are the estimated geometric mean IC50 from the meta-regression (described in the methods). Closed circles indicate that the geometric mean IC50 was above 10,000 ng/ml and error bars indicate the 95% Confidence intervals of the geometric mean IC50. The geometric mean neutralization titer of serum collected from a cohort of convalescent individuals was reported in a subset of studies in the Stanford University Coronavirus and Resistance database, and these

are shown (blue) along with the geometric mean neutralization titer from the meta-regression. The fitted model and confidence intervals were derived from a global model fit of n=802 unique observations.

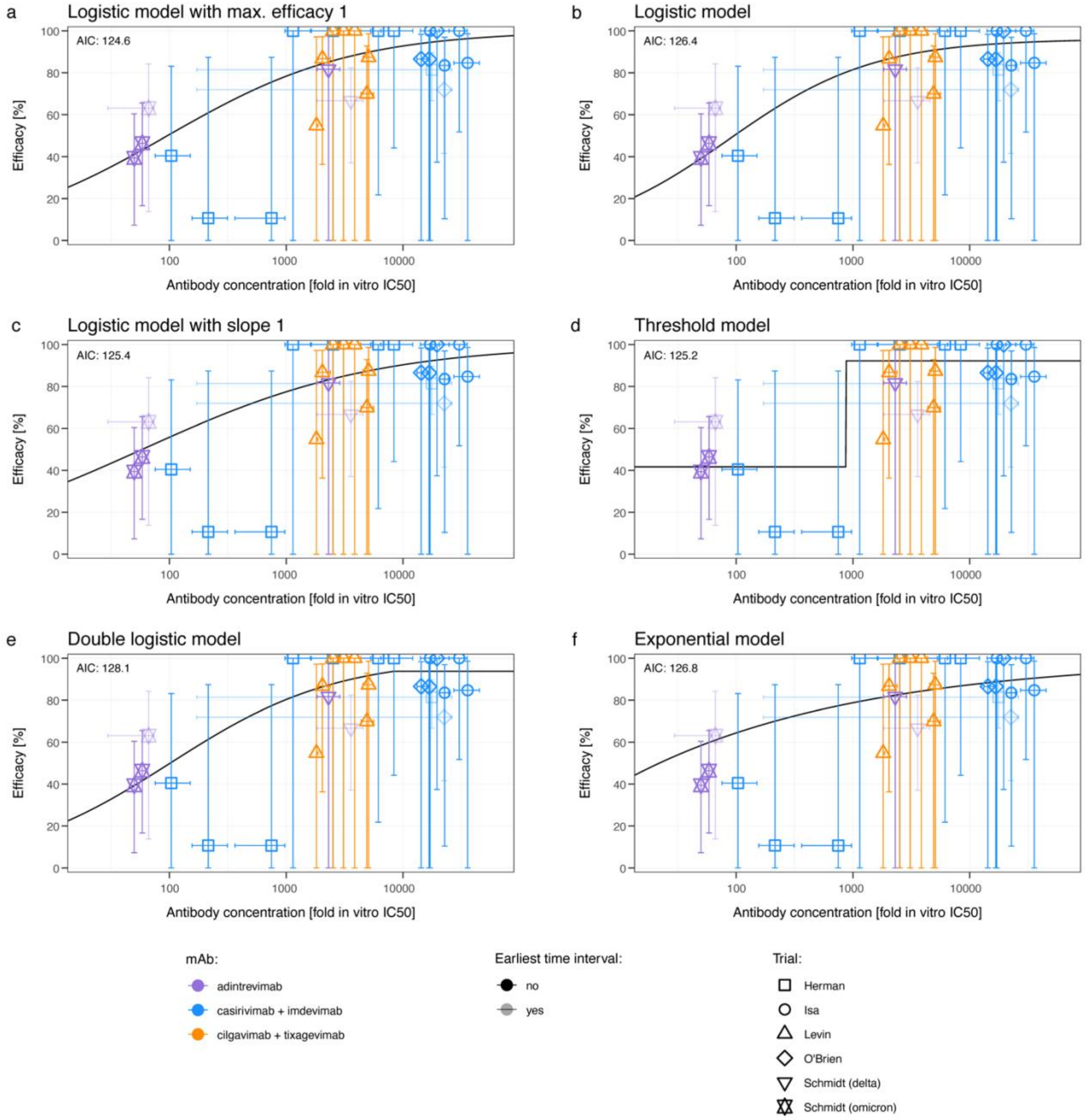

Figure S2 Different efficacy functions fit to prophylactic mAb treatment data. The efficacy models that were fit to the data are the logistic model with maximum 1 (**a**), the logistic model (**b**), the logistic model with slope parameter 1 (**c**), a threshold model (**d**), a double logistic model (**e**), and an exponential model (**f**). The different models and their AICs are also specified in Table S5. All panels contain a fitted model (black line) to n=24 unique data points. Vertical error bars indicating the 95% confidence interval of the observations and horizontal error bars indicate the maximum and minimum (mean) antibody concentrations observed during each time interval.

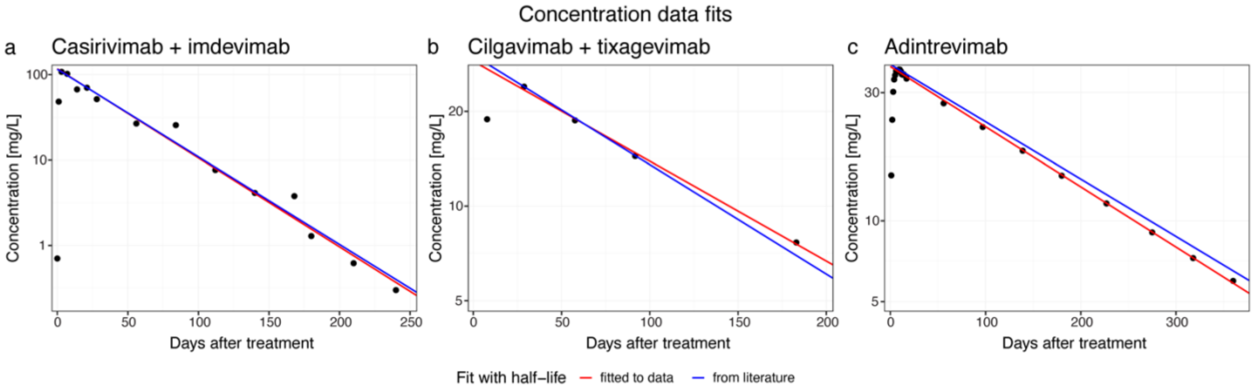

Figure S3 Antibody concentration data extracted from O'Brien et al.[8] and Herman et al.[9] (**a**), Levin et al.[7] (**b**) and Schmidt et al.[10] (**c**) for casirivimab/imdevimab (**a**), tixagevimab/cilgavimab (**b**), and adintrevimab (**c**), respectively (black dots). Also shown are the best fitting line (linear regression) to the data from the peak reported concentration to the end of the time series (red). The half-life for each antibody combination reported in the literature is shown in blue for comparison (see also Table S8).

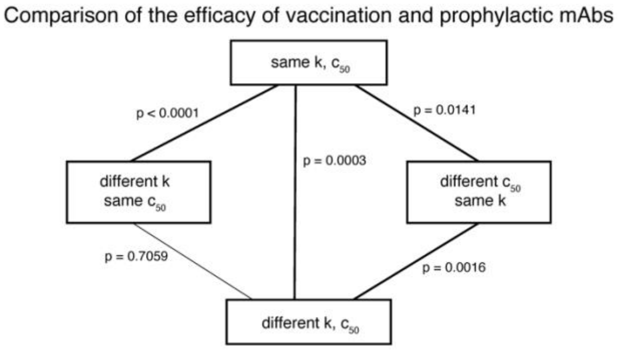

Figure S4 Comparison of model fits to the vaccination and mAb efficacy data using a forward regression strategy. To compare the efficacy of vaccination and prophylactic mAb treatment, we fitted a logistic dose-response curve with maximal efficacy 1 to the data (Figure 4b and Figure S5). The parameters of the dose-response curve are a slope parameter (k) and the neutralization titer (on the fold of convalescence scale) that gives 50% protection (c_{50}). First, we fitted the dose-response curve to all data and assumed the model parameters are the same for vaccines and mAbs ("same k, c_{50}", see Figure S5). Next, we allowed a single parameter to differ between the vaccines and mAbs and compared the resulting model with the first model using a likelihood-ratio test (indicated p-values). This showed the biggest improvement by allowing the slope to vary (see red and blue lines in Figure 4b). We fitted one further model allowing both the slope and the neutralization titer that gives 50% protection to vary between both types of treatment. We compared the models using a likelihood ratio test (see p-values above) and the AIC (see Table S9). We found the best fit is the model that allows for different slopes but has the same 50% protection neutralization for vaccines and mAbs (red and blue lines in Figure 4b). The parameter values for different model fits can be found in Table S9.

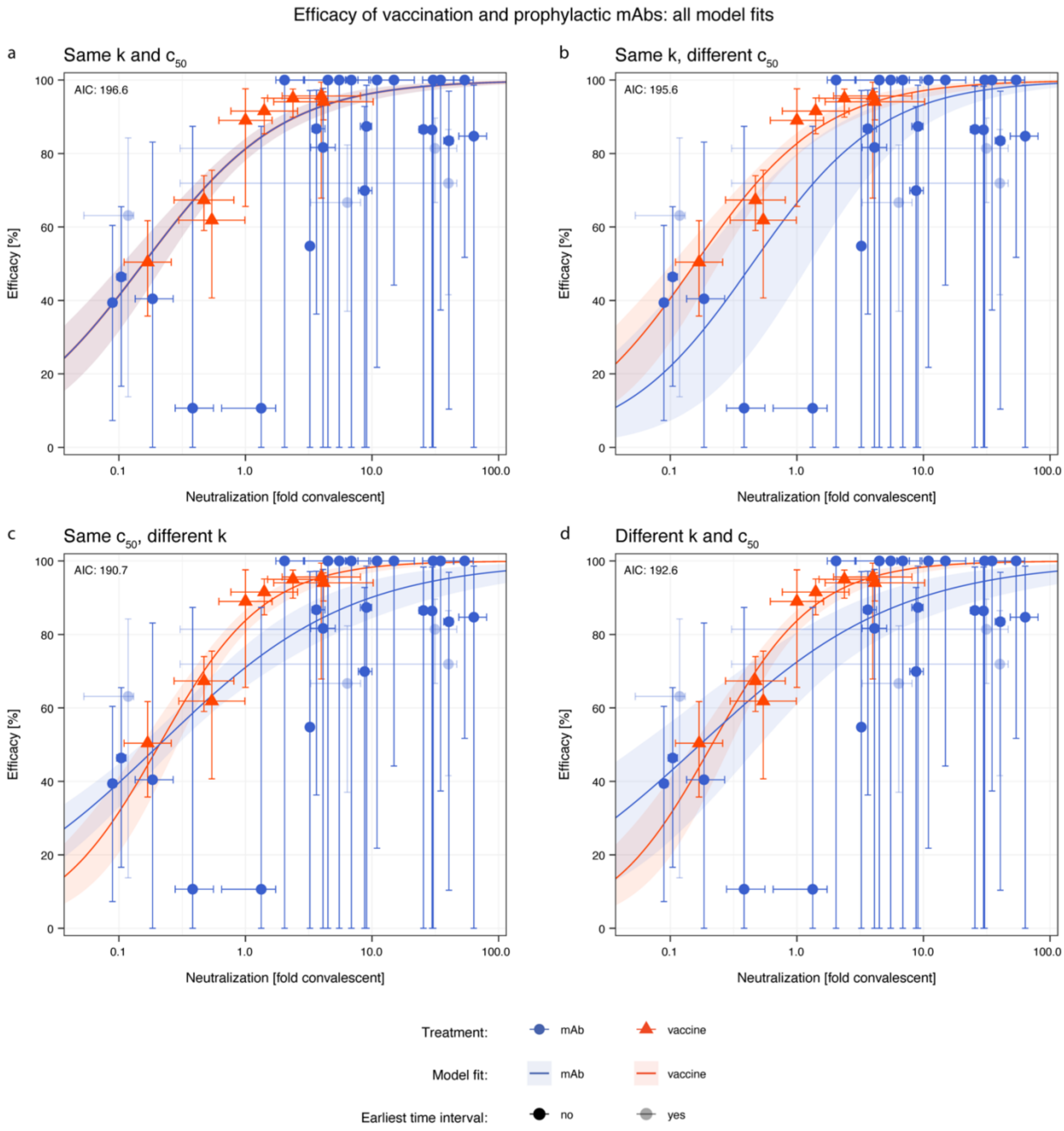

Figure S5 Different models fit to the prophylactic mAb treatment and vaccination data. We fit a logistic efficacy model with maximal efficacy 1 simultaneously to the mAb (blue) (n=24 unique data points) and vaccine (red) data (n=8 unique data points). The parameters of the model are the slope parameter and the neutralization titer that gives 50% protection. Each panel shows a model fit where these parameters are either the same or different for mAbs and vaccines: (**a**) same slope parameter (k) and neutralization titer giving 50% protection (c_{50}), (**b**) same k but different c_{50}, (**c**) same c_{50} but different k, and (**d**) different k and different c_{50}. The parameter values and AICs for each model can be found in Table S9. Vertical error bars indicate the 95% confidence intervals (CI) of the efficacy, and horizontal error bars indicate the maximum and minimum (mean) neutralizing antibody titre observed during each time interval (blue) or 95% CI of the mean neutralizing antibody titre (red).

References

1. Tzou PL, Tao K, Pond SLK, Shafer RW. Coronavirus Resistance Database (CoV-RDB): SARS-CoV-2 susceptibility to monoclonal antibodies, convalescent plasma, and plasma from vaccinated persons. *PLoS One* **17**, e0261045 (2022).

2. Vaida F, Fitzgerald AP, Degruttola V. Efficient Hybrid EM for Linear and Nonlinear Mixed Effects Models with Censored Response. *Comput Stat Data Anal* **51**, 5718-5730 (2007).

3. Vaida F, Liu L. Fast Implementation for Normal Mixed Effects Models With Censored Response. *J Comput Graph Stat* **18**, 797-817 (2009).

4. Rohatgi A. WebPlotDigitizer - Web Based Plot Digitizer. https://apps.automeris.io/wpd/ (Version 4.6).

5. Adobe Inc. Adobe Illustrator. Available at https://adobe.com/products/illustrator (2022).

6. Isa F*, et al.* Repeat subcutaneous administration of casirivimab and imdevimab in adults is well-tolerated and prevents the occurrence of COVID-19. *Int J Infect Dis* **122**, 585-592 (2022).

7. Levin MJ*, et al.* Intramuscular AZD7442 (Tixagevimab-Cilgavimab) for Prevention of Covid-19. *N Engl J Med* **386**, 2188-2200 (2022).

8. O'Brien MP*, et al.* Subcutaneous REGEN-COV Antibody Combination to Prevent Covid-19. *N Engl J Med* **385**, 1184-1195 (2021).

9. Herman GA*, et al.* Efficacy and safety of a single dose of casirivimab and imdevimab for the prevention of COVID-19 over an 8-month period: a randomised, double-blind, placebo-controlled trial. *Lancet Infect Dis* **22**, 1444-1454 (2022).

10. Schmidt P*, et al.* Antibody-mediated protection against symptomatic COVID-19 can be achieved at low serum neutralizing titers. *Sci Transl Med* **15**, eadg2783 (2023).

11. Stadler E*, et al.* Determinants of passive antibody efficacy in SARS-CoV-2 infection. *medRxiv*, doi.org/10.1101/2022.03.21.22272672 (2022).

12. Aho K, Bowyer RT. Confidence intervals for ratios of proportions: implications for selection ratios. *Methods in Ecology and Evolution* **6**, 121-132 (2015).

13. Khoury DS*, et al.* Neutralizing antibody levels are highly predictive of immune protection from symptomatic SARS-CoV-2 infection. *Nat Med* **27**, 1205-1211 (2021).

14. R Core Team. R: A Language and Environment for Statistical Computing. *R Foundation for Statistical Computing* **Vienna, Austria**, https://www.R-project.org/ (2019).

15. Bates D, Mächler M, Bolker B, Walker S. Fitting Linear Mixed-Effects Models Using lme4. *Journal of Statistical Software* **67**, 1 - 48 (2015).

16. Rubin D. Chapter 2: Multiple imputation. In: *Flexible Imputation of Missing Data (Second Edition)* (Edited by: van Buuren S). Chapman and Hall (2018).

17. Hothorn T, Bretz F, Westfall P. Simultaneous inference in general parametric models. *Biom J* **50**, 346-63 (2008).

18. Regeneron Pharmaceuticals Inc. Emergency Use Authorization (EUA) of REGEN-COV (casirivimab and imdevimab). https://www.regeneron.com/downloads/treatment-covid19-eua-fact-sheet-for-hcp.pdf (accessed 13 Oct 2022) (2022).

19. Australian Product Information. EVUSHELD tixagevimab and cilgavimab. https://www.tga.gov.au/sites/default/files/evusheld-pi.pdf (accessed 13 Oct 2022) (2022).
