# Supplementary material for: Monoclonal antibody levels and protection from COVID-19
Source: Nat Commun. 2023 Jul 28;14:4545. doi: 10.1038/s41467-023-40204-1 (PMC10382502; doi:10.1038/s41467-023-40204-1)
Supplement: Supplementary file 3 — Reporting Summary [file 41467_2023_40204_MOESM3_ESM.pdf]

nature portfolio

Corresponding author(s): David Khoury

Last updated by author(s): Jul 6, 2023

Reporting Summary

Nature Portfolio wishes to improve the reproducibility of the work that we publish. This form provides structure for consistency and transparency in reporting. For further information on Nature Portfolio policies, see our Editorial Policies and the Editorial Policy Checklist.

Statistics

For all statistical analyses, confirm that the following items are present in the figure legend, table legend, main text, or Methods section.

n/a	Confirmed	
☐	☒	The exact sample size (*n*) for each experimental group/condition, given as a discrete number and unit of measurement
☒	☐	A statement on whether measurements were taken from distinct samples or whether the same sample was measured repeatedly
☐	☒	The statistical test(s) used AND whether they are one- or two-sided<br>*Only common tests should be described solely by name; describe more complex techniques in the Methods section.*
☐	☒	A description of all covariates tested
☐	☒	A description of any assumptions or corrections, such as tests of normality and adjustment for multiple comparisons
☐	☒	A full description of the statistical parameters including central tendency (e.g. means) or other basic estimates (e.g. regression coefficient) AND variation (e.g. standard deviation) or associated estimates of uncertainty (e.g. confidence intervals)
☐	☒	For null hypothesis testing, the test statistic (e.g. *F*, *t*, *r*) with confidence intervals, effect sizes, degrees of freedom and *P* value noted<br>*Give P values as exact values whenever suitable.*
☒	☐	For Bayesian analysis, information on the choice of priors and Markov chain Monte Carlo settings
☒	☐	For hierarchical and complex designs, identification of the appropriate level for tests and full reporting of outcomes
☐	☒	Estimates of effect sizes (e.g. Cohen's *d*, Pearson's *r*), indicating how they were calculated

Our web collection on statistics for biologists contains articles on many of the points above.

Software and code

Policy information about availability of computer code

Data collection	All data was collected from published studies randomised controlled trials of monoclonal antibodies used to prevent COVID-19. Data was extracted manually, or with WebPlotDigitizer [Rohatgi A. WebPlotDigitizer - Web Based Plot Digitizer. https://apps.automeris.io/wpd/ (Version 4.6)].
Data analysis	Data analysis was performed in the R statistical software package (Version 4.2.1). All data analysis code and tools are available publicly on GitHub at https://github.com/david-s-khoury/COVID19-mAb-prophylaxis

For manuscripts utilizing custom algorithms or software that are central to the research but not yet described in published literature, software must be made available to editors and reviewers. We strongly encourage code deposition in a community repository (e.g. GitHub). See the Nature Portfolio guidelines for submitting code & software for further information.

Data

Policy information about availability of data

All manuscripts must include a data availability statement. This statement should provide the following information, where applicable:

- Accession codes, unique identifiers, or web links for publicly available datasets
- A description of any restrictions on data availability
- For clinical datasets or third party data, please ensure that the statement adheres to our policy

All data are available publicly on GitHub at https://github.com/david-s-khoury/COVID19-mAb-prophylaxis.

Data was obtained from publicly available sources:
- Stanford Coronavirus Antiviral and Resistance Database (https://covdb.stanford.edu/)

- Isa F, et al. Repeat subcutaneous administration of casirivimab and imdevimab in adults is well-tolerated and prevents the occurrence of COVID-19. Int J Infect Dis 122, 585-592 (2022).

- Levin MJ, et al. Intramuscular AZD7442 (Tixagevimab-Cilgavimab) for Prevention of Covid-19. N Engl J Med 386, 2188-2200 (2022).

- O'Brien MP, et al. Subcutaneous REGEN-COV Antibody Combination to Prevent Covid-19. N Engl J Med 385, 1184-1195 (2021).

- Herman GA, et al. Efficacy and safety of a single dose of casirivimab and imdevimab for the prevention of COVID-19 over an 8-month period: a randomised, double-blind, placebo-controlled trial. Lancet Infect Dis 22, 1444-1454 (2022).

- Schmidt P, et al. Antibody-mediated protection against symptomatic COVID-19 can be achieved at low serum neutralizing titers. Sci Transl Med 15, eadg2783 (2023).

Research involving human participants, their data, or biological material

Policy information about studies with human participants or human data. See also policy information about sex, gender (identity/presentation), and sexual orientation and race, ethnicity and racism.

Reporting on sex and gender	NA - meta-analysis - only aggregate data from each study was available
Reporting on race, ethnicity, or other socially relevant groupings	NA - meta-analysis - only aggregate data from each study was available
Population characteristics	Population characteristics, regarding the immunization status of of individuals, risk factors for COVID and sero-status are summarized in Table S1 and Table S2.
Recruitment	NA - meta-analysis - no participants were directly recruited in this study.
Ethics oversight	This work was approved under the UNSW Sydney Human Research Ethics Committee (approval HC200242).

Note that full information on the approval of the study protocol must also be provided in the manuscript.

Field-specific reporting

Please select the one below that is the best fit for your research. If you are not sure, read the appropriate sections before making your selection.

[x] Life sciences [ ] Behavioural & social sciences [ ] Ecological, evolutionary & environmental sciences

For a reference copy of the document with all sections, see nature.com/documents/nr-reporting-summary-flat.pdf

Life sciences study design

All studies must disclose on these points even when the disclosure is negative.

Sample size	Sample size was determined by the primary studies as described within each published study.
Data exclusions	Studies were excluded if they did not meeting inclusion criteria of systematic search (detailed in supplementary material). Of included studies all data was extracted and included in analysis. For some particular analysis any data that was excluded (e.g. in leave-one-out analysis) is explained in the text.
Replication	All extracted data was verified by two authors, and the concordance of extracted data was compared directly and found to be highly concordant.
Randomization	All studies included in this meta-analysis were randomized controlled trials and followed their own randomization procedure, in our study subjects were not reallocated to other groupings, and thus no further randomization of subjects was performed in this study.
Blinding	All studies included in this meta-analysis were randomized controlled trials and followed their own blinding procedure - no blinding occurred in this study, since no randomization or reallocation of subjects was performed in our study.

Reporting for specific materials, systems and methods

We require information from authors about some types of materials, experimental systems and methods used in many studies. Here, indicate whether each material, system or method listed is relevant to your study. If you are not sure if a list item applies to your research, read the appropriate section before selecting a response.

Materials & experimental systems

n/a	Involved in the study
☒	☐ Antibodies
☒	☐ Eukaryotic cell lines
☒	☐ Palaeontology and archaeology
☒	☐ Animals and other organisms
☒	☐ Clinical data
☒	☐ Dual use research of concern
☒	☐ Plants

Methods

n/a	Involved in the study
☒	☐ ChIP-seq
☒	☐ Flow cytometry
☒	☐ MRI-based neuroimaging
